# Shear Wave Elastography for Characterization of Breast Lesions in Clinical Routine

**DOI:** 10.1002/jum.70231

**Published:** 2026-03-09

**Authors:** Sylvia Kelemen, Tician Schnitzler, Simin Laures, Daniela Schwegler‐Guggemos, Nikki Rommers, Sebastian Schindera, Michael Golatta

**Affiliations:** ^1^ Faculty of Medicine University of Basel Basel Switzerland; ^2^ Cantonal Hospital Aarau Institute of Radiology Aarau Switzerland; ^3^ Department of Clinical Research University of Basel Basel Switzerland; ^4^ Breast Cancer Center Klinikum Sankt Elisabeth Heidelberg Germany; ^5^ University Breast Unit, Department of Obstetrics and Gynecology Heidelberg University Hospital Heidelberg Germany

**Keywords:** BI‐RADS, breast cancer, diagnostic accuracy, shear wave elastography, ultrasound

## Abstract

**Objectives:**

Breast cancer remains a major global health challenge, and conventional B‐mode ultrasound frequently encounters limitations in reliably distinguishing benign from malignant lesions, particularly within intermediate Breast Imaging‐Reporting and Data System (BI‐RADS) categories. These uncertainties often contribute to unnecessary biopsies. Shear wave elastography (SWE), a quantitative method for assessing tissue stiffness, may improve diagnostic confidence by providing objective elasticity thresholds. This study aimed to evaluate the diagnostic accuracy of SWE in routine clinical practice and to determine optimal stiffness cut‐off values for differentiating benign from malignant breast lesions.

**Methods:**

In this prospective single‐center study, 73 women (mean age 48.6 ± 15.6 years) with BI‐RADS 3–5 breast lesions underwent SWE examination prior to biopsy. Stiffness measurements (kPa and m/s) were obtained for each lesion. Histopathology served as the reference standard. Diagnostic performance was assessed, and optimal thresholds were determined using receiver‐operating characteristic (ROC) analysis. Intra‐ and interobserver reproducibility were evaluated.

**Results:**

Malignant lesions demonstrated significantly higher stiffness values (mean 56.7 kPa) compared with benign lesions (mean 18.7 kPa). SWE exhibited excellent diagnostic accuracy with an AUC of 0.932. The optimal cut‐off value for distinguishing malignant from benign lesions was 40.03 kPa (sensitivity 88%, specificity 95%), corresponding to 3.6 m/s. Stiffness measurements showed high reproducibility across observers.

**Conclusion:**

SWE accurately differentiates benign from malignant breast lesions, improving specificity, and potentially reducing unnecessary biopsies. Standardization is needed to support widespread clinical adoption.

**Abbreviations**
AI: artificial intelligence
AUC: area under the curve
BI‐RADS: Breast Imaging‐Reporting and Data System
DEGUM: Deutsche Gesellschaft für Ultraschall in der Medizin
IQR: interquartile range
kPa: kilopascal
ROC: receiver‐operating characteristic
ROI: region of interest
SWE: shear wave elastography

Breast cancer is the most common malignancy among women worldwide and remains a major public health challenge. Mammography and ultrasound are the primary imaging modalities for breast cancer detection and characterization. However, conventional ultrasound often fails to reliably distinguish between benign and malignant breast lesions, particularly in intermediate Breast Imaging Reporting and Data System (BI‐RADS) categories, which leads to diagnostic uncertainty and frequently necessitates histologic confirmation through biopsy. These additional procedures contribute to patient anxiety and increased healthcare costs.

Shear wave elastography (SWE) is an advanced ultrasound technique that quantifies tissue stiffness by measuring the propagation velocity of shear waves induced by acoustic radiation forces from the transducer.^1^, ^2^, ^3^ Malignant tissues are typically stiffer than normal parenchyma and exhibit higher elasticity values, measurable in kilopascals (kPa) or meters per second (m/s).^4^ SWE provides a color‐coded map that visually represents tissue stiffness, thereby improving lesion characterization and reducing subjectivity in interpretation.^5^ Studies have demonstrated low interobserver variability^6^ and enhanced diagnostic specificity without loss of sensitivity, making SWE a valuable adjunct to conventional ultrasound.^7^

Despite its proven potential, SWE is not yet universally implemented as a standard diagnostic tool.^8^ Technical variability among manufacturers and a lack of standardized acquisition parameters remain major obstacles to broader clinical adoption.^9^, ^10^ Additionally, the absence of universally accepted cutoff values for differentiating benign from malignant lesions limits reproducibility and comparability across studies. Nonetheless, SWE has been incorporated into the most recent BI‐RADS edition, underscoring its growing relevance in breast imaging.^11^ Professional societies, including the German Society for Ultrasound in Medicine (DEGUM), also recommend SWE as a complementary diagnostic method.^12^

This study aimed to evaluate the diagnostic accuracy of SWE using the Canon Aplio i700 ultrasound system (Canon Medical Systems, Otawara, Japan). In addition, we compared our findings with the only published study utilizing the same device^13^ to determine an optimal cutoff value for distinguishing benign from malignant breast lesions in our patient cohort and to validate these results against existing literature using the same as well as different devices. The goal was to provide further evidence supporting the routine clinical adoption of SWE in breast cancer diagnostics.

## Materials and Methods

### Study Design and Patient Population

This prospective, observational single‐center study was approved by the Swiss Association of Research Ethics Committees (BASEC ID 2022‐00087). The study was conducted at a tertiary care hospital between May 2022 and December 2023. Female patients aged 18 years or older with BI‐RADS 3, 4, or 5 lesions identified on imaging who subsequently underwent biopsy were eligible. Biopsies of BI‐RADS 3 lesions were performed exclusively at the patient's request, in line with BI‐RADS recommendations, according to which BI‐RADS 3 lesions warrant imaging surveillance, whereas BI‐RADS 4 and 5 lesions warrant biopsy.

Exclusion criteria included prior ipsilateral breast malignancy, presence of breast implants, pregnancy, breastfeeding, or lack of informed consent. For patients with multiple lesions undergoing biopsy, the lesion with the lowest BI‐RADS category was included in the analysis. This criterion applied to 9 patients.

### Ultrasound Examination

SWE was performed using Aplio i700 ultrasound systems (Canon Medical Systems Corporation; software version 3.1, updated to 4.0 during the study with identical SWE settings). The systems were equipped with 7–18 MHz linear transducers. Three devices standardized for this protocol were used. Elastography was performed using a circular region of interest (ROI) with a 2 mm diameter.

**Figure 1:**
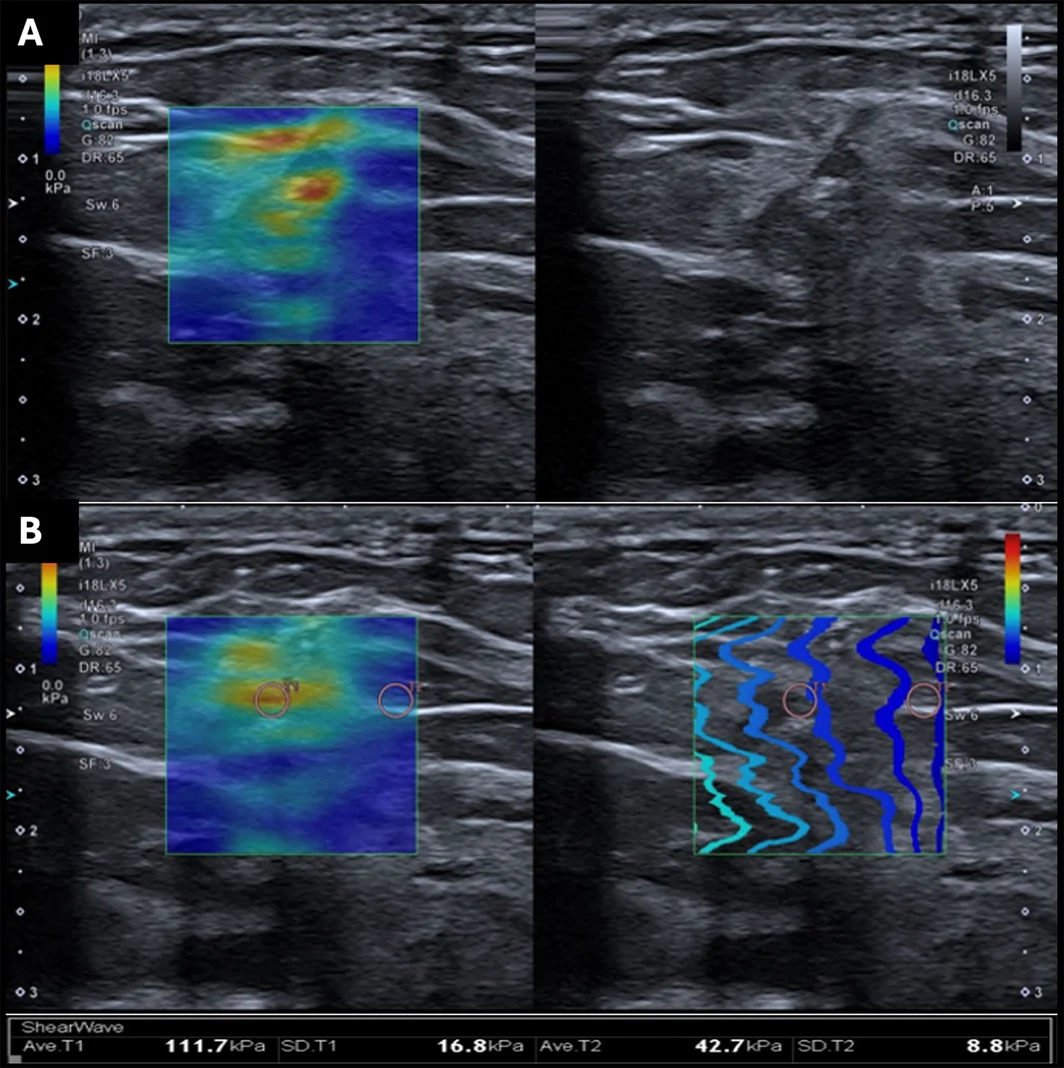
A 41‐year‐old woman with breast mass. Splitscreen image with (**A**) shear wave on the left and B‐mode on the right and (**B**) with measurements on the left and propagation map on the right. Histology confirmed invasive carcinoma.

**Figure 2:**
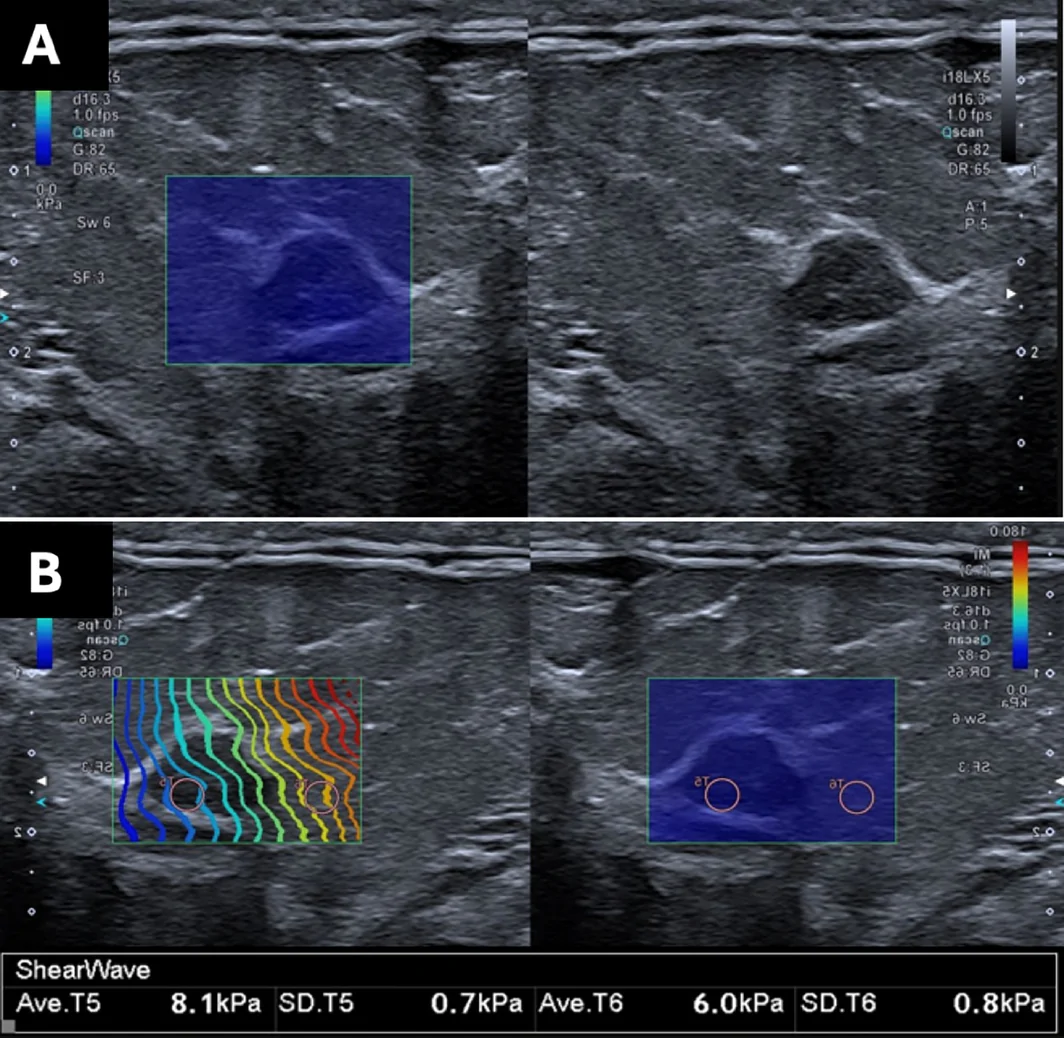
A 48‐year‐old woman with breast mass. Splitscreen image with (**A**) shear wave on the left and B‐mode on the right and (**B**) with measurements on the right and propagation map on the left. Histology confirmed fibroadenoma.

B‐mode imaging was first obtained in 2 perpendicular planes to measure lesion dimensions. The optimal plane was then selected for SWE acquisition. The sampling box was adjusted to encompass the entire lesion whenever possible. Images were acquired in split‐screen mode to display both the propagation map and the lesion for quality control (Figures 1 and 2). Three consecutive measurements were obtained, targeting the stiffest visible lesion area. Adjacent tissue at comparable depth was also measured within the same field. All SWE images and measurements were saved as DICOM files. Lesions were categorized using the fifth edition of the BI‐RADS Atlas before SWE acquisition.

### Data Collection

All examinations were performed by 4 breast radiologists with 3.5, 5, 7, and 16 years of experience in breast imaging, respectively. The Aplio i700 system had been available since June 2019, and all radiologists had prior experience with the device. To ensure procedural consistency, all operators received standardized training and written instructions and the technical settings of the device (eg, frequency and ROI size) were stored as a preset. Only measurements acquired using the correctly selected preset were included to ensure reproducibility. Of the included examinations, 8 were performed by TS, 38 by SK, 8 by SL, and 19 by DSG.

Lesion stiffness (in kPa and m/s) and parenchymal stiffness (in kPa) were recorded. Diagnostic accuracy was determined using histologic results as the reference standard. Receiver‐operating characteristic (ROC) curve analysis was used to identify the optimal stiffness threshold for differentiating malignant from benign lesions. Sensitivity, specificity, and accuracy were calculated accordingly.

Shear wave velocity (Vs) and elasticity (E) are related by the formula *E =* 3ρVs^2^.^14^ The ultrasound device used reported stiffness in kPa; for comparability, values were also expressed in m/s using this relationship.

### Statistical Analysis

All enrolled patients were included in the full analysis set. Baseline characteristics were summarized by lesion type (benign versus malignant) using mean ± standard deviation or median (interquartile range) for continuous variables and counts (percentages) for categorical variables.

ROC curve analysis was performed using the mean of 3 stiffness measurements to determine the optimal diagnostic cutoff. Histopathology served as the gold standard. The best threshold was defined as the value yielding the highest diagnostic accuracy with a minimum sensitivity of 60%. The area under the curve (AUC) was computed with the DeLong method.^15^ Sensitivity and specificity were reported with bootstrap‐based 95% confidence intervals^16^; diagnostic accuracy was estimated using Blaker's method.^17^

Measurement reproducibility was evaluated by calculating within‐patient variation across the 3 SWE acquisitions. The Pearson correlation coefficient was used to assess the relationship between lesion depth and stiffness.

## Results

### Patient and Lesion Characteristics

A total of 73 female patients with BI‐RADS 3, 4, or 5 lesions were included (Table 1). The mean patient age was 48.6 years (SD, 15.6). Patients with benign lesions were significantly younger (mean, 44.7 years; SD, 14.0) than those with malignant lesions (mean, 61.5 years; SD, 13.8). Histopathologic evaluation revealed 56 benign (76.7%) and 17 malignant (23.3%) lesions. All B3 lesions (*n* = 3) underwent surgical excision and were classified as 2 papillomas and 1 fibroadenoma.

**Table 1:** Patient and Lesion Characteristics

	All (*n* = 73)	Benign (*n* = 56)	Malignant (*n* = 17)
Age, mean (SD)	48.62 (15.56)	44.71 (13.95)	61.47 (13.83)
Lesion width, median [IQR]	10.30 [7.50, 14.10]	9.00 [5.70, 13.72]	12.70 [9.90, 18.60]
Lesion height, median [IQR]	6.40 [4.20, 9.10]	5.45 [4.00, 7.80]	9.10 [6.90, 12.20]
Lesion length, median [IQR]	10.00 [6.60, 13.50]	9.60 [6.22, 12.17]	10.40 [9.80, 18.50]
Maximal lesion diameter, median [IQR]	10.80 [8.70, 14.10]	10.25 [8.05, 13.72]	12.70 [10.30, 19.80]
Mean lesion stiffness (kpa), median [IQR]	20.10 [11.90, 39.97]	18.70 [10.55, 24.45]	56.70 [44.10, 88.00]
Mean lesion stiffness (m/s), median [IQR]	2.59 [2.00, 3.63]	2.50 [1.87, 2.84]	4.33 [3.78, 5.40]
Mean lesion depth (mm), median [IQR]	12 [0.83, 1.53]	11.7 [0.79, 1.48]	14.3 [1.10, 2.13]
Mean parenchymal stiffness (kPa), median [IQR]	8.90 [6.60, 11.90]	7.78 [6.20, 10.53]	12.83 [10.90, 16.63]
Mean parenchymal stiffness (m/s), median [IQR]	1.72 [1.48, 1.99]	1.61 [1.44, 1.87]	2.07 [1.91, 2.35]
BI‐RADS score			
3	6 (8.2%)	6 (10.7%)	0 (0.0%)
4a	43 (58.9%)	43 (76.8%)	0 (0.0%)
4b	7 (9.6%)	6 (10.7%)	1 (5.9%)
4c	6 (8.2%)	0 (0.0%)	6 (35.3%)
5	11 (15.1%)	1 (1.8%)	10 (58.8%)
Histology			
Carcinoma in situ	1 (1.4%)	0 (0.0%)	1 (5.9%)
Fibroadenoma	31 (42.5%)	31 (55.4%)	0 (0.0%)
Fibrosis and fibrocystic changes	9 (12.3%)	9 (16.1%)	0 (0.0%)
Invasive breast cancer	16 (21.9%)	0 (0.0%)	16 (94.1%)
Other benign entities	14 (19.2%)	14 (25.0%)	0 (0.0%)
Papilloma	2 (2.7%)	2 (3.6%)	0 (0.0%)

Malignant lesions were larger across all measured dimensions, with a median maximum diameter of 12.7 mm (interquartile range [IQR], 10.3–19.8), compared with 10.2 mm (IQR, 8.0–13.7) for benign lesions. Median lesion stiffness was substantially higher in malignant lesions (56.7 kPa [IQR, 44.1–88.0]; 4.3 m/s [IQR, 3.8–5.4]) than in benign lesions (18.7 kPa [IQR, 10.5–24.4]; 2.5 m/s [IQR, 1.9–2.8]). Mean parenchymal stiffness was also higher in malignant lesions (12.8 kPa [IQR, 10.9–16.6]; 2.7 m/s) compared with benign lesions (7.8 kPa [IQR, 6.2–10.5]; 1.6 m/s) (Table 2).

**Table 2:** Summary Statistics of the 3 Measurements

	Overall (*n* = 73)
Lesion stiffness 1 (kpa), median [IQR]	20.60 [12.80, 39.90]
Lesion stiffness 2 (kpa), median [IQR]	20.40 [11.70, 36.60]
Lesion stiffness 3 (kpa), median [IQR]	20.20 [11.40, 35.40]
Lesion depth 1 (mm), median [IQR]	1.20 [0.80, 1.50]
Lesion depth 2 (mm), median [IQR]	1.20 [0.90, 1.70]
Lesion depth 3 (mm), median [IQR]	1.20 [0.80, 1.60]
Parenchymal stiffness 1 (kpa), median [IQR]	8.40 [5.80, 12.30]
Parenchymal stiffness 2 (kpa), median [IQR]	8.60 [6.30, 13.50]
Parenchymal stiffness 3 (kpa), median [IQR]	8.10 [6.30, 11.80]

### Diagnostic Performance of Lesion Stiffness

**Figure 3:**
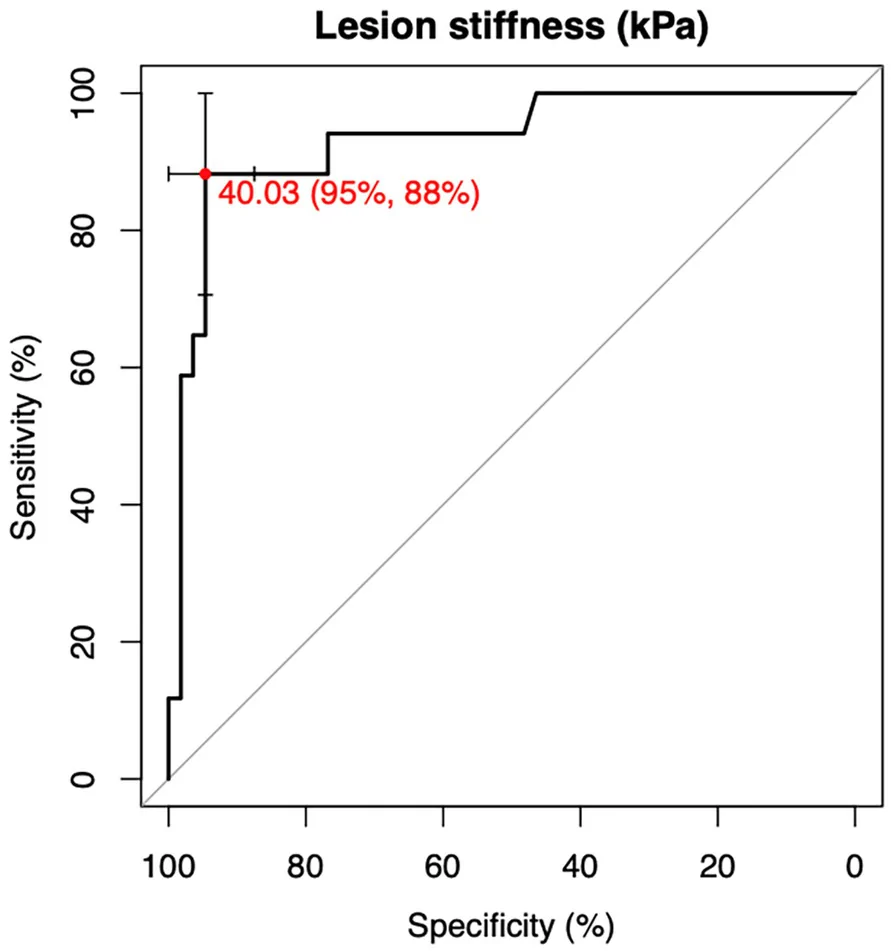
ROC curve for lesion stiffness measured in kPa. In red, the threshold value (sensitivity, specificity).

**Figure 4:**
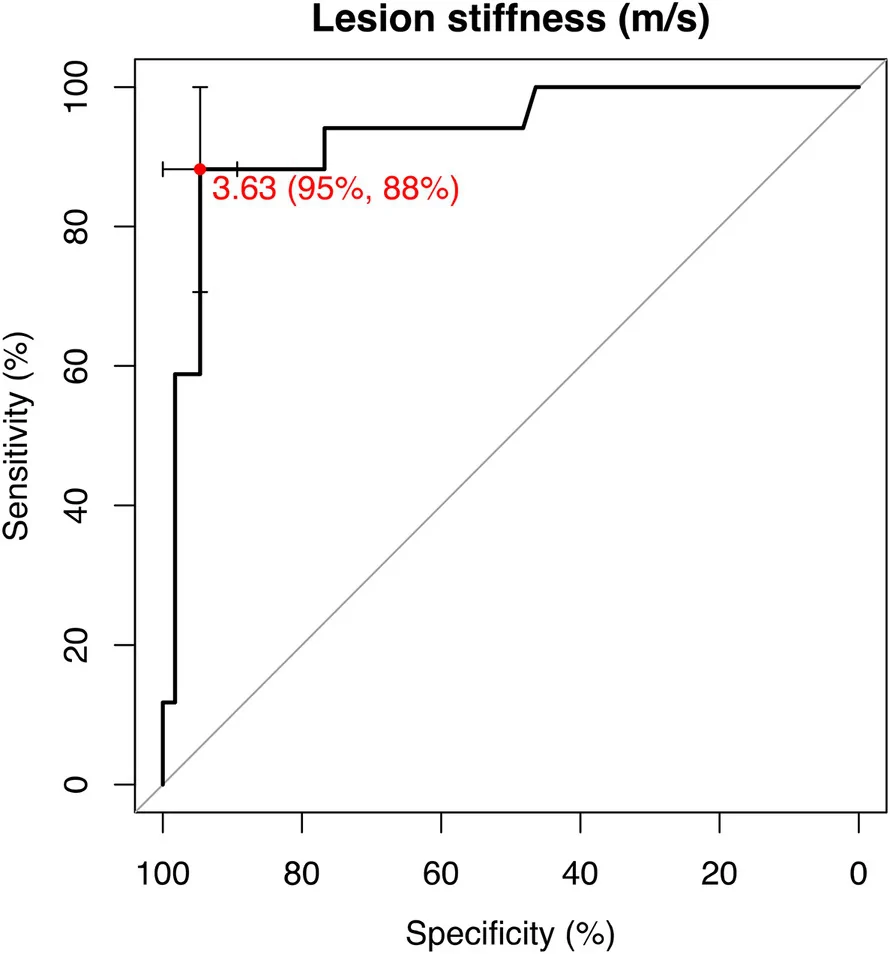
ROC curve for lesion stiffness measured in m/s. In red, the threshold value (sensitivity, specificity).

**Figure 5:**
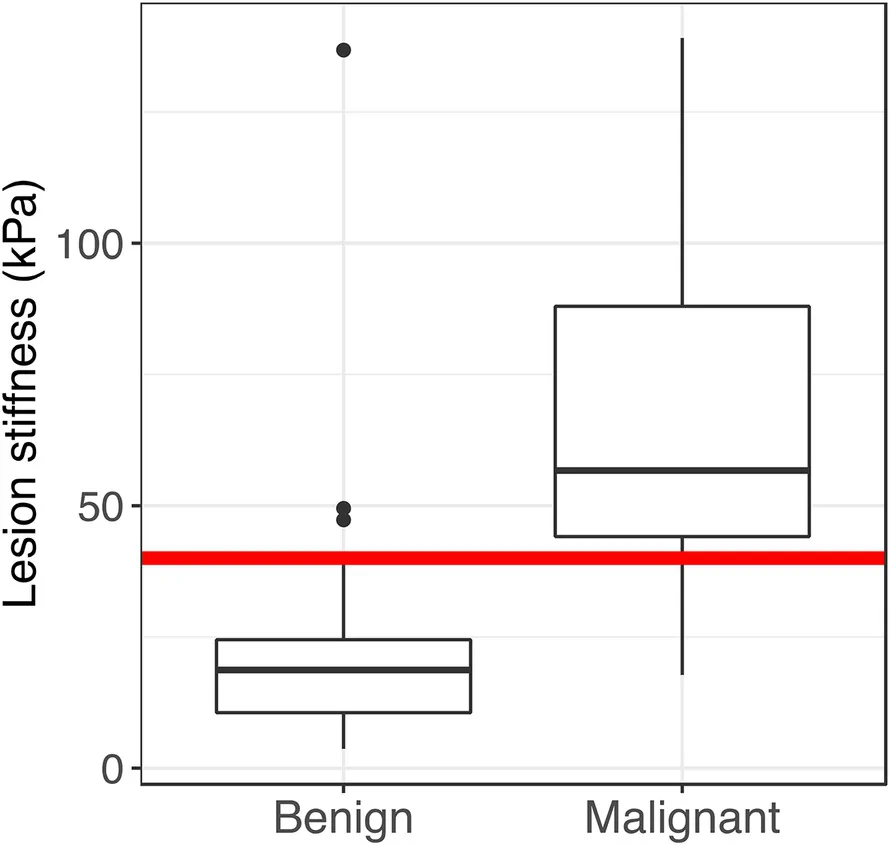
Boxplot showing the distribution of lesion stiffness in the benign and malignant group. The red line represents the optimal cut‐off in kPa identified through the ROC analysis.

**Figure 6:**
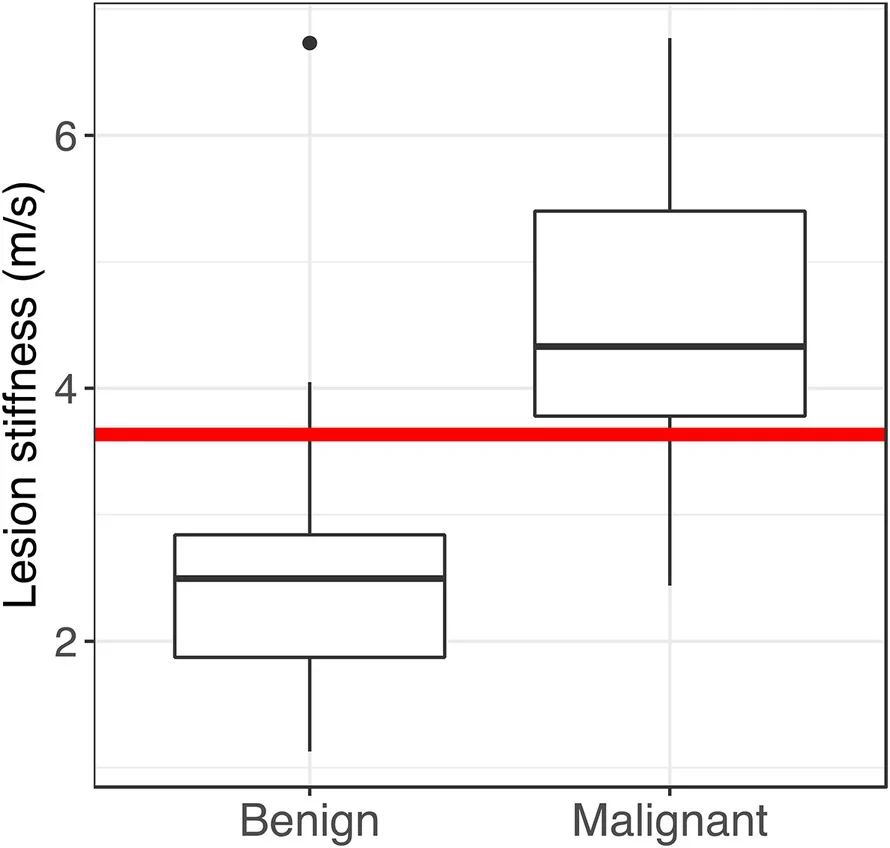
Boxplot showing the distribution of lesion stiffness in the benign and malignant group. The red line represents the optimal cut‐off in m/s identified through the ROC analysis.

ROC curve analysis demonstrated excellent diagnostic discrimination of lesion stiffness for differentiating malignant from benign lesions (Table 3). The area under the ROC curve (AUC) was 0.932 (95% CI, 0.861–1.000) for stiffness in kilopascals (Figure 3) and 0.931 (95% CI, 0.860–1.000) for stiffness in meters per second (Figure 4). The optimal cutoff value of 40.0 kPa (3.6 m/s) yielded a sensitivity of 88% (95% CI, 71–100), specificity of 95% (95% CI, 88–100), and overall diagnostic accuracy of 93% (95% CI, 85–97) (Figures 5 and 6).

**Table 3:** Diagnostic Performance of Lesion Stiffness (kPa and m/s) for Distinguishing Malignant and Benign Lesions

Measure	kPa	m/s
AUC	0.932 [0.861, 1.000]	0.931 [0.860, 1.000]
Best threshold	40	3.6
Sensitivity (%)	88 [71, 100]	88 [71, 100]
Specificity (%)	95 [88, 100]	95 [88, 100]
Accuracy (%)	93 [85, 97]	93 [85, 97]

Area under the receiver‐operating characteristic curve (AUC), optimal thresholds, sensitivity, specificity, and accuracy with 95% confidence intervals (CI) are presented.

### Reproducibility of SWE Measurements

The reproducibility of SWE measurements was high. The median within‐patient variation for lesion stiffness was 4.1 kPa (IQR, 2.3–10.3) and 1.17 m/s, respectively. Parenchymal stiffness values demonstrated similarly low variability, and lesion depth measurements exhibited consistent reproducibility (median difference, 0.2 mm; IQR, 0.1–0.3).

### Correlation Between Stiffness and Lesion Characteristics

Correlation analysis showed no significant association between lesion stiffness and lesion depth, confirming that SWE measurements were stable across varying depths and lesion sizes.

### Exploratory Analyses

**Figure 7:**
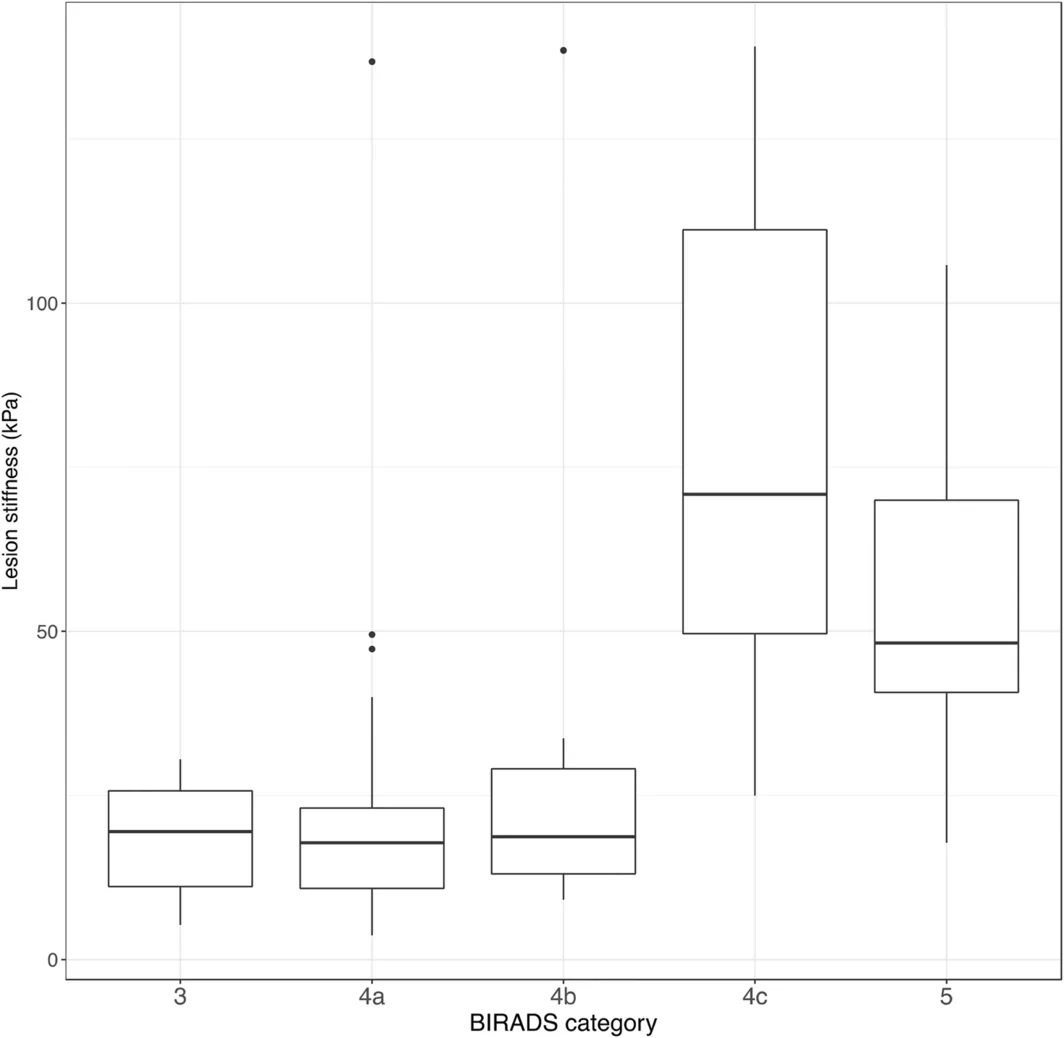
Boxplots of lesion stiffness (in kPa) by BI‐RADS category.

Lesion stiffness increased progressively with higher BI‐RADS categories, particularly in BI‐RADS 4C and 5 lesions (Figure 7). Median stiffness values were significantly higher in BI‐RADS 5 lesions compared with lower categories, consistent with the increased likelihood of malignancy in these groups. Greater variability in stiffness was observed among BI‐RADS 4C and 5 lesions, reflecting the heterogeneity of tissue composition in highly suspicious or malignant cases.

While deeper lesions exhibited slightly greater within‐patient variability in stiffness, overall diagnostic performance was unaffected. SWE demonstrated consistent stiffness estimates across varying lesion depths, supporting its robustness in different anatomical contexts.

## Discussion

This study evaluated the diagnostic performance of SWE using the Canon Aplio i700 ultrasound system (Canon Medical Systems, Otawara, Japan) for differentiating benign from malignant breast lesions using histopathology as the reference standard. The results demonstrate that SWE provides excellent diagnostic accuracy, with lesion stiffness emerging as a reliable quantitative parameter for malignancy detection. These findings support the integration of SWE into breast ultrasound protocols, particularly for indeterminate BI‐RADS categories where diagnostic uncertainty often leads to unnecessary biopsies.

The ROC analysis revealed an AUC of 0.932, confirming SWE's strong discriminative ability. The optimal cutoff value of 40.0 kPa (3.6 m/s) achieved high sensitivity (88%) and specificity (95%). Using this cutoff, 55 biopsies would have been avoided, at the cost of missing 2 invasive carcinomas and 3 lesions that were ultimately confirmed as fibroadenomas (all categorized as BI‐RADS 4a) would still have been biopsied. The other lesions above this cutoff were classified as BI‐RADS 4b (1 lesion), 4c (5 lesions), or 5 (9 lesions) and were histologically proven invasive carcinomas. Accordingly, the negative predictive value would be 96.4% and the positive predictive value was 85.7%. This demonstrates the potential of SWE to reduce unnecessary biopsies and also supports BI‐RADS recommendations to consider SWE as a complementary tool to BI‐RADS assessment rather than a standalone method.

Our cutoff in general aligns with previously reported values, whereas other investigations using different vendors' systems have described cutoff values ranging from 50 to 81 kPa.^7^, ^18^, ^19^ These differences likely persist due to variation in equipment and acquisition protocols. The only study to our knowledge using the same ultrasound system reported a higher optimal cutoff of 86.5 kPa (5.37 m/s).^13^ The small sample size in our study may have contributed to the observed differences in cutoff values for the same device. Additionally, differences in patient populations, as the other study was conducted in Korea, may have influenced the results as well.

Such variation underscores the need for vendor‐specific standardization and harmonized SWE acquisition protocols.

Parenchymal stiffness was also higher in malignant lesions, consistent with prior work suggesting that peritumoral stroma may undergo fibrotic or desmoplastic changes.^20^, ^21^ Although absolute stiffness values differed among studies, the observed trend—higher parenchymal stiffness in malignancy—remained consistent across different ultrasound systems.

Measurement reproducibility in our cohort was excellent, with minimal intra‐observer variability. This finding corroborates earlier reports demonstrating SWE's robustness and low interobserver variability.^6^ High reproducibility is critical for longitudinal follow‐up, lesion characterization, and multi‐institutional implementation.

Exploratory analysis revealed a stepwise increase in lesion stiffness with higher BI‐RADS categories, particularly in BI‐RADS 4C and 5. However, no malignant lesions were identified in the BI‐RADS 4A subgroup, likely reflecting the limited sample size. A single benign lesion categorized as BI‐RADS 5 was histologically confirmed as a papilloma with usual ductal hyperplasia, illustrating the occasional discordance between imaging and pathology and the ongoing necessity of histologic confirmation in high‐suspicion cases.

From a clinical standpoint, SWE offers the potential to reduce the number of benign biopsies by improving diagnostic specificity. Quantitative stiffness measurement provides an objective complement to conventional B‐mode assessment, particularly in intermediate BI‐RADS lesions. This aligns with current efforts to refine ultrasound‐based risk stratification through quantitative imaging biomarkers.^22^

Emerging evidence suggests that SWE can also assist in evaluating axillary lymph nodes^23^, ^24^ and in monitoring treatment response in patients receiving neoadjuvant chemotherapy.^25^ Furthermore, studies integrating artificial intelligence (AI) with SWE have shown promising results, enabling a substantial reduction in unnecessary biopsies without compromising sensitivity.^26^

Despite these advantages, several challenges remain. Inter‐vendor variability continues to hinder cross‐study comparability, and no universally accepted cutoff value exists. Standardization efforts—similar to those that facilitated the widespread adoption of liver elastography—will be essential for SWE to become a routine component of breast imaging.^27^ Importantly, our findings indicate that even when the same device is used, optimal cutoff values may differ slightly across studies, likely reflecting differences in patient populations. Additionally, multicenter studies with larger sample sizes are required to validate threshold values and confirm reproducibility across platforms.

This study has limitations. The relatively small sample size and single‐center design may limit generalizability. Furthermore, the unequal distribution of cases among radiologists reflects routine clinical workflow but may have introduced minor operator‐related variability.

Another limitation of our study is the small number of BI‐RADS 3 lesions, reflecting the nature of our prospective design in routine clinical practice, where these lesions are typically managed with short‐term imaging follow‐up rather than biopsy, which was performed only at the patient's request. The relatively small sample size in some subgroups may limit the statistical power.

Overall, SWE demonstrated excellent diagnostic performance, high reproducibility, and strong potential to reduce unnecessary biopsies. These findings reinforce SWE's role as a reliable adjunct to conventional ultrasound in breast cancer diagnostics.

## Conclusions

SWE demonstrated excellent diagnostic performance in differentiating benign from malignant breast lesions, with high reproducibility and robustness across lesion sizes and depths. Quantitative stiffness measurement provides valuable complementary information to conventional ultrasound, enhancing diagnostic specificity and potentially reducing unnecessary biopsies.

Although inter‐vendor variability and the absence of universally accepted cutoff values continue to limit widespread adoption, the results of this study support SWE as a reliable adjunct in routine breast ultrasound. Standardization of acquisition parameters and multicenter validation studies are needed to facilitate cross‐platform comparability. Integration of advanced analytic methods, including artificial intelligence, may further strengthen SWE's role in precision breast imaging.

## Data Availability

The data that support the findings of this study are available from the corresponding author upon reasonable request.
